# Triptolide Induces hepatotoxicity via inhibition of CYP450s in Rat liver microsomes

**DOI:** 10.1186/s12906-016-1504-3

**Published:** 2017-01-05

**Authors:** Yan Lu, Tong Xie, Yajie Zhang, Fuqiong Zhou, Jie Ruan, Weina Zhu, Huaxu Zhu, Zhe Feng, Xueping Zhou

**Affiliations:** 1 The First Clinical College, Nanjing University of Chinese Medicine, Nanjing, Jiangsu China; 2 The Third Affiliated Hospital, Nanjing University of Chinese Medicine, Nanjing, Jiangsu China; 3 College of Pharmacy, Nanjing University of Chinese Medicine, Nanjing, Jiangsu China

**Keywords:** CYP450s, Hepatotoxicity, Microsomes, Triptolide

## Abstract

**Background:**

Triptolide (TP), an active constituent of Tripterygium wilfordii, possesses numerous pharmacological activities. However, its effects on cytochrome P450 enzymes (CYP450s) in rats remain unexplored.

**Methods:**

In this study, the effects of triptolide on the six main CYP450 isoforms (1A2, 2C9, 2C19, 2D6, 2E1, and 3A) were investigated both in vivo and in vitro. We monitored the body weight, survival proportions, liver index, changes in pathology, and biochemical index upon TP administration, in vivo. Using a cocktail probe of CYP450 isoform-specific substrates and their metabolites, we then carried out in vitro enzymatic studies in liver microsomal incubation systems via ultra-high performance liquid chromatography-tandem mass spectrometry (UHPLC-MS/MS). Finally, we verified our results at the messenger ribonucleic acid (mRNA) and protein level through quantitative real-time polymerase chain reaction (RT-qPCR), western blotting, and immunohistochemical detection.

**Results:**

The in vivo toxicity study confirmed that Sprague-Dawley (SD) rats exhibited dose-dependent hepatotoxicity after intragastric administration of TP [200, 400, and 600 μg/(kg.day)] for 28 days. In case of the CYP450 isoforms 3A, 2C9, 2C19, and 2E1, the in vitro metabolic study demonstrated a decrease in the substrate metabolic rate, metabolite production rate, and Vmax, with an increase in the Km value, compared with that observed in the control group. Additionally, a TP dose-dependent decrease in the mRNA levels was observed in the four major isoforms of CYP3A subfamily (3A1/3A23, 3A2, 3A9, and 3A62) and CYP2C9. A similar effect was also observed with respect to the protein levels of CYP2C19 and CYP2E1.

**Conclusions:**

This study suggests that TP can cause hepatotoxicity by reducing the substrate affinity, activity, and expression at the transcriptional and protein levels of the CYP450 isoforms 3A, 2C9, 2C19, and 2E1. TP also has the potential to cause pharmacokinetic drug interactions when co-administered with drugs metabolized by these four isoforms. However, further clinical studies are needed to evaluate the significance of this interaction.

**Electronic supplementary material:**

The online version of this article (doi:10.1186/s12906-016-1504-3) contains supplementary material, which is available to authorized users.

## Background

**Fig. 1:**
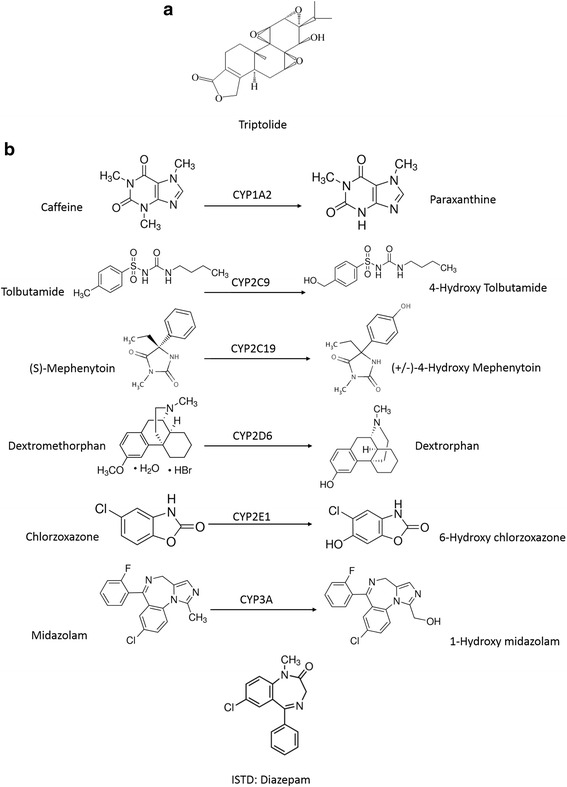
Related chemical structures. **a** The chemical structure of TP. **b** The chemical structure of the cocktail probe

Triptolide (TP) (Fig. 1a) is a major bioactive diterpenoid isolated from the traditional Chinese medicinal herb *Tripterygium wilfordii Hook F* (TWHF). It shows promising anti-inflammatory, immunomodulatory, anti-proliferative, proapoptotic, and neuroprotective activities [1–6]. However, its clinical application is limited owing to acute and chronic side effects induced in multiple organs. According to the China Food and Drug Administration (CFDA), commercial preparations of T. wilfordii were responsible for 633 adverse reaction cases from 2004 to 2011 September, including 53 severe cases that involved reproductive toxicity, hepatotoxicity, and renal cytotoxicity among other outcomes [7]. The mechanism underlying to TP-induced liver injury is caused by many reasons. In L-02 cells, TP decreased mitochondrial membrane potential and Bcl-2, promoted the release of cytochrome c, and up-regulated the expression of Bax, P53 and caspase 3 via the mitochondrial apoptotic pathway [8]. It was also reported that TP treatment significantly increased ROS levels and decreased GSH levels, decreased the protein expression of Nrf2 and its target genes including HO-1 and MRP2 except NQO [9]. Recent research also indicates the abnormal metabolism of Cytochrome P450s (CYP450s) enzyme system plays an important role.

Drug-induced toxicity is caused either by the parent compounds or by their reactive metabolites generated primarily through biotransformation in the liver [10]. CYP450s, a monooxygenase family with comprehensive detoxification, which play a dominant role in the redox metabolism of numerous endobiotics [11] and xenobiotics [12], are closely associated with the toxic effects of TP [13, 14]. According to a previous study, TP was absorbed, distributed, and monohydroxylated rapidly in the liver after oral administration [15]. Further, in vitro data indicated that the metabolism of TP was mediated by both CYP3A4 and CYP2C19 [16]. Additionally, dexamethasone, a CYP3A agonist, was observed to markedly accelerate the metabolism of TP and thus attenuate hepatotoxicity in vivo [17]. TP is also reported to inhibit CYP3A4 expression and activity [18]. Thus, the CYP3A subfamily has been reported to play an important role in TP-induced hepatotoxicity [18, 19].

However, the underlying mechanisms of TP-induced toxicity mediated by the CYP450 family have not been well delineated. Since abnormal drug metabolism by CYP450s also plays an important role in the hepatotoxicity, the enzymes might trigger drug-drug interactions (DDIs) between TP and other co-administered drugs, thereby, causing the observed toxicity. In this study, we focused on understanding the role of CYP450s in mediating TP-induced hepatotoxicity. We demonstrated that TP disturbed the activity and expression of CYP450s in the liver, through UHPLC-MS/MS, RT-qPCR, and western blot analyses. We also evaluated the TP-induced in vivo hepatotoxicity using several physiological and biochemical indices. Our study focused on the six main isoforms of CYP450s: 1A2, 2C9, 2C19, 2D6, 2E1, and 3a. These six isoforms account for more than 80% of the hepatic CYP450s, and metabolize more than 90% of the clinically approved drugs [20]. The substrates, metabolites in the cocktail probe used in this study are shown in (Fig. 1b). Based on the results obtained, we concluded that CYP3A, CYP2C9, CYP2C19, and CYP2E1 were the CYP450 subfamily members most affected by TP.

## Methods

### Chemicals and reagents

TP (CAS No. 38748-32-2), tolbutamide (D860), midazolam (MDZ), chlorzoxazone (CLZ), caffeine (CAF), paraxanthine, 4-hydroxytolbutamide, ±−−4-hydroxymephenytoin, dextrorphan, 1-hydroxymidazolam, diazepam, NADPH, D-glucose-6-phosphate disodium salt, and glucose-6-phosphate dehydrogenase were purchased from Sigma-Aldrich (St. Louis, MO, USA). S-mephenytoin (MT) and 6-hydroxychlorzoxazone were purchased from Santa Cruz Biotechnology (Santa Cruz, CA, USA). Dextromethorphan (DM) was obtained from the National Institute for the Control of Pharmaceutical and Biological Products (Beijing, China). High-performance liquid chromatography-grade acetonitrile, methanol, formic acid, and ethyl acetate were obtained from Merck (Darmstadt, Germany). Deionized water was obtained using a Milli-Q® system (Millipore Corporation, Billerica, MA). Quantitative detection kits for Alanine aminotransferase (ALT)、aspartate aminotransferase (AST)、total protein (Tp)、globulin(GLB)、albumin(ALB)、triglycerides (TG)、total cholesterol (T-chol)、and glucose (Glu) were obtained in Beijing Leadman Biochemistry Technology Co. Ltd (Beijing China). Carbon monoxide was obtained from Nanjing Weichuang Gas Co. Ltd. (Nanjing, China). Radioimmunoprecipitation assay buffer, phenylmethanesulfonyl fluoride (PMSF), and sodium dodecyl sulfate-polyacrylamide gel electrophoresis (SDS-PAGE) sample loading buffer were purchased from Beyotime Institute of Biotechnology (Jiangsu, China). Polyvinylidene difluoride (PVDF) membranes were obtained from Millipore (Shanghai, China). Rabbit anti-rat CYP2C19 polyclonal antibody and rabbit anti-rat CYP2E1 polyclonal antibody were purchased from Abcam (Cambridge, MA, USA). The bicinchoninic acid (BCA) protein assay kit was purchased from Thermo Fisher Scientific (Waltham, MA, USA). All other chemicals and solvents used were of analytical grade.

### Animals and experimental design

Since the toxic effects of TP are significantly different between different sexes [21], we specifically chose female SD rats for our study. All animal studies were performed with the approval of the Institutional Animal Care and Use Committee of Nanjing University of Chinese Medicine, and were performed according to the guidelines of the National Institutes of Health for the Care and Use of Laboratory Animals (NIH publication No. 80–23). Female SD rats (180–220 g) were purchased from the Charles River Experimental Animal Company (Beijing, China). All rats were housed in an air-conditioned animal quarter at a temperature of 23–27 °C and a relative humidity of 45–55%, with *ad libitum* access to water and food. The animals were acclimatized to the facilities for a week and then fasted, with free access to water for 12 h prior to each experiment. The rats were randomly divided into four groups of 12 animals each. While the control group rats received the vehicle alone, the three TP group rats received different concentrations of TP (200 μg/(kg.day), 400 μg/(kg.day), and 600 μg/(kg.day), once daily for 28 days. During the experiment, the rats were generally monitored on a daily basis and their body weights were measured weekly. After the final drug administration, the rats were fasted overnight, anesthetized with 10% chloral hydrate (0.35 mL/100 g) by intraperitoneal injection, and then sacrificed. Blood samples were drawn and the livers were collected for further analysis.

### In vivo toxicity study

The liver samples were immediately weighed for liver index calculation (liver weight × 100%/body weight) and frozen in liquid nitrogen. Abdominal aortic blood was drawn and centrifuged at 3000 rpm for 5 min (4 °C) to collect the blood serum for the determination of ALT, AST, Tp, ALB, TG, T-chol, and GLU levels by using an automatic biochemistry analyzer (LX20, Beckman Coulter Inc., Miami, FL, USA). Slices of the same part of the livers were cut and fixed in phosphate-buffered 10% formaldehyde solution and then embedded in paraffin wax. Sections of liver tissue were cut and stained with hematoxylin and eosin and examined for histopathological changes under an Olympus DX45 microscope (Olympus Corporation, Tokyo, Japan). The images were taken using an Olympus DP72 camera (Olympus Corporation, Tokyo, Japan) at an original magnification of 200 × .

### In vitro metabolic study

#### Preparation and appraisal of rat liver microsomes

Rat liver microsomes were obtained via differential centrifugation [22] (L-80XP, Microfuge® 16, Beckman Coulter Inc., Miami, FL, USA). The microsomes were stored in buffer [10 mM Tris-acetate, 1 mM ethylenediaminetetraacetic acid (EDTA), 100 mM PMSF, and 20% glycerol, pH: 7.4] at −80 °C until further use. Protein concentrations were determined by the BCA method and CYP450 content was determined by the method of Omura and Sato [23].

#### Study of CYP450 activity in in vitro microsomal incubation systems

The enzymatic activities of P450 isoforms were characterized based on the reactions shown in (Fig. 1b). The microsomal incubation system (200 μL) consisted of a buffer [10% sucrose, 100 mM EDTA, 1 mM PMSF, 2 mM dithiothreitol (DTT), and 100 mM phosphate-buffered saline (PBS), pH 7.4], an NADPH regeneration system (0.2 mM NADP+, 2 mM glucose-6-phosphate, and 0.4 IU glucose-6-phosphate dehydrogenase), hepatic microsomes [0.5 mg (protein)/mL], an inhibitor, and a cocktail probe (CAF/D860/MT/DM/CLZ/MDZ: 15/20/10/10/5/2.5 μM) [24].

After pre-incubation in a shaking incubator block (Eppendorf Thermomixer 5436, Hamburg, Germany) for 5 min at 37 °C the reaction was initiated by adding NADP+ (0.2 mM). The reactions were performed at 37 °C for 30 min and terminated by the addition of 200 μL of ice-cold methanol - acetonitrile (1: 1, V: V) solution, which contained Diazepam (8 μg/mL) as an internal standard (ISTD). The mixtures were immediately cooled in an ice bath to precipitate the proteins, then shaken and centrifuged for 10 min at 12000 rpm. An aliquot of 20 μL of the supernatant was injected for UHPLC–MS/MS analysis to test the final concentration of substrates and metabolites. The substrate metabolic rate (%) and the production of the metabolites [nmol/(protein g × min)] was used as the enzyme activity index.

To estimate the kinetic parameters, the microsomes were incubated with different concentrations of the cocktail probe (diluted by half dilution method as listed in Table 1). The concentration of each metabolite was determined by UHPLC-MS/MS at different timepoints (1, 2, 4, 6, and 10 min) and the metabolic rate was calculated by linear regression analysis. The Vmax and Km value of each CYP450 isoform was calculated by non-linear regression analysis of experimental data according to the Michaelis–Menten model of enzyme kinetics, using the Prism 5.0 program:

**Table 1:** The cocktail probe of different concentration (μM)

Substrates	Concentration Number						
	1	2	3	4	5	6	7
Caffeine(CAF)	1.875	3.75	7.5	15	30	60	120
Tolbutamide(D860)	0.625	1.25	2.5	5	10	20	40
(S)- Mephenytoin(MT)	1.25	2.5	5	10	20	40	60
Dextromethorphan(DM)	2.5	5	10	20	40	80	160
Chlorzoxazone(CLZ)	1.25	2.5	5	10	20	40	60
Midazolam(MDZ)	0.313	0.625	1.25	2.5	5	10	20

$$ V=\frac{V_{\max}\left[S\right]}{\left({K}_m+\left[S\right]\right)} $$

Where V is the metabolic rate; Vmax is the maximum velocity; Km is the Michaelis–Menten constant; [S] is the substrate concentration.

#### UHPLC–MS/MS-ESI analysis

Chromatographic separations were carried out on an Agilent 1290 UHPLC system (Agilent Technologies, Santa Clara, CA, USA) using an Agilent ZORBAX RRHD Eclipse Plus C18 column (2.1 × 50 mm inner diameter,1.8 μm particle size) (Agilent Technologies, Santa Clara, CA, USA). The elution profile was composed of an initial isocratic step with water (0.1% formic acid): acetonitrile (ACN) (95:5) for 1.5 min and then increasing ACN to 30% over 4 min to separate the substrates and metabolites at 30 °C. The column was washed with 90% of ACN for 2.5 min followed by the equilibration of the column for 5 min with 5% ACN. The flow rate was 0.4 mL/min and the injection volume was 5 μL. An Agilent 6460 triple quadrupole-mass spectrometer with an electrospray ion source (Agilent Technologies, Santa Clara, CA, USA) was used for flow injection analysis with optimized fragmentor and source parameters. The optimized source parameters for MS analysis were drying gas temperature, 350 °C; gas flow, 10 L/min; nebulizer gas flow pressure, 35 psi; and capillary voltage, 4500 V.

### Verification of observed effects at the mRNA and protein level

#### Quantitative real-time polymerase chain reaction

While implementing this experiment, we realized that human CYP3A subfamily isoforms, such as CYP3A4, CYP3A43, CYP3A7, etc., have several commercially available antibodies; however, there are no such antibodies for the main CYP3A subfamily isoforms (e.g. CYP3A1, CYP3A2, etc.) and CYP2C9 of rats. In order to validate the metabolic study and further probe the specific roles of the CYP3A subfamily and CYP2C9, we measured the mRNA expression of five major CYP3A subfamily isoforms (CYP3A1/3A23, CYP3A2, CYP3A9, CYP3A18, and CYP3A62) [25–28] and CYP2C9 using RT-qPCR. Frozen tissue samples were homogenized in 1 mL Trizol® reagent and total RNA was extracted following the manufacturer’s protocol. After ethanol precipitation, the vacuum-dried RNA was dissolved in 50 μL nuclease-free water, and the concentration and purity of isolated RNA was determined by absorbance at 260 nM. The 260/280 nM ratios of the samples were between 1.8 and 2.0. RNA purity and concentration was assessed using a NanoDrop 2000 spectrophotometer (Thermo Fisher Scientific Waltham, MA, USA). Thus, cDNA synthesis was performed at 50 °C for 60 min by adding 7 μL of the RT master mix (“PrimeScriptTM RT Master Mix RR036A (TaKaRa, Tokyo, Japan)) and terminated by incubating for 15 min at 75 °C. Distilled water was added to the reaction mixture to a final volume of 100 μL. Real-time qPCR was performed with an ABI® 7500 system (Life Technologies Corporation, Gaithersburg, MD, USA) using the 2x SYBR® Select Master Mix (Thermo Fisher Scientific, Waltham, MA, USA) for the detection of PCR products. β-actin was used as an internal control. The primer pairs used were as listed in Table 2. Amplification was done with a hot start polymerase activation step for 10 min at 95 °C, followed by 40 cycles of 15 s at 95 °C and 1 min at 60 °C, and the cycle threshold (CT) values of the target gene were identified. All samples were run in triplicates.

**Table 2:** Primers used in RT-PCR

Target gene	Sequence of primers	
CYP3A1/3A23	forward	TTTCCTTTGTCCTGCATTCC
	reverse	CATAGGTGGGAGGTGCCTTA
CYP3A2	forward	CGTCGATTCCCTTAACAACC
	reverse	AATCCTTTGGGAACATGCAG
CYP3A9	forward	ACAAAGACCCGCATTACTGG
	reverse	GCAAACCTCATGCCAATACA
CYP3A18	forward	CCAATAAGGCACCTGTCACC
	reverse	AGGGTTCCGATGAAGAGGAT
CYP3A62	forward	GATGTGGAGATTGTGGCTCA
	reverse	TCTGGAGATCAGGGTGTGTG
CYP2C9	forward	CTGCTGCTGCTGAAACACGTG
	reverse	GGATGACAGCGTACTATCAC
β-actin	forward	TCACCCACACTGTGCCCATCTATGA
	reverse	CATCGGAACCGCTCATTGCCGATAG

#### Western blotting

The total protein extract was prepared by diluting 50 μL liver microsome suspension with 1 volume of radioimmunoprecipitation assay (RIPA) lysis buffer (150 mM NaCl, 1% Nonidet™ P-40 (NP-40), 0.5% sodium deoxycholic acid, 0.1% SDS, and 50 mM Tris-Cl, pH 7.5) for 15 min and then centrifuging at 13,000 rpm 4 °C for 10 min. Aliquots containing 20 μg of protein were separated by SDS-PAGE on a 10% gradient gel (Invitrogen, Carlsbad, CA, USA) and transferred to a PVDF membrane which was blocked with 5% non-fat dry milk in Tris-buffered saline with Tween 20 (TBST) (200 mM Tris–HCl, 1.37 M NaCl, 0.1% Tween 20, pH 7.6) for 1 h. The membrane was then incubated with rabbit anti-rat CYP2C19 polyclonal antibody (1:2000 dilution), rabbit anti-rat CYP2E1 polyclonal antibody (1:1000 dilution) and glyceraldehyde-3-phosphate dehydrogenase (GAPDH) antibody (1:2000 dilution), overnight at 4 °C. After rinsing five times with TBST, for 5 min each time, at 20 °C, the membrane was incubated with horseradish peroxidase (HRP)-labeled anti-rabbit immunoglobulin G (IgG) antibody (Santa Cruz Biotechnology, Santa Cruz, CA, USA). The immunoreactive bands were visualized using an enhanced chemiluminescence detection system (ChemiDoc™ XRS+, Bio-Rad, Hercules, CA, USA) and quantified by densitometry using ImageJ 1.41 (National Institutes of Health, Bethesda, Maryland, USA) software.

#### Immunohistochemical detection

The tissue sections were incubated with rabbit anti-rat CYP2C19 polyclonal antibody (1:200 dilution) and rabbit anti-rat CYP2E1 polyclonal antibody (1:100 dilution) in blocking solution (0.4% (v/v) Triton X-100 and 3% (v/v) normal goat serum (NGS) in PBS) for 1 h. After incubation, the slices were washed four times, for 10 min each time, with 0.01 M PBS. Then, the sections were again incubated for 2 h with anti-rabbit antibody (1:500 dilution) at room temperature. After several PBS washes, the antibody complex was detected using a modification of the ABC system (Vectastain® ABC kit, Vector Laboratory Inc., Burlingame, CA, USA). The immunoreactive cells were stereologically quantified using Image-Pro Express 6 (Media Cybernetics, CA, USA) software. The selected areas were digitized with an Olympus DX45 microscope (Olympus Corporation, Tokyo, Japan). The images were taken using an Olympus DP72 camera (Olympus Corporation, Tokyo, Japan) at an original magnification of 400 × .

### Statistical analysis

SPSS version 19.0 for Windows (IBM Corp, Armonk, NY, USA) was used for all statistical analyses. All experiments were performed at least thrice. Comparisons were made between two groups using two-sided Student’s *t* test and among multiple groups using one-way ANOVA followed by Tukey’s post hoc test. Data are expressed as Mean ± SD and *P* < 0.05 was considered statistically significant.

## Results

### In vivo toxicity study

#### General observation and survival proportions

**Fig. 2:**
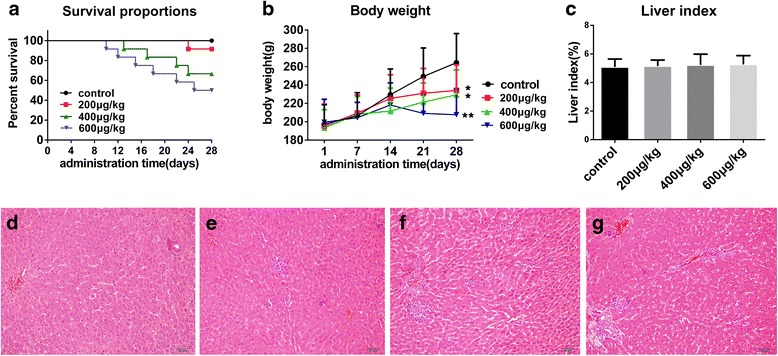
General and pathological study in vivo. **a** Survival proportions during oral administration of TP of different dosage. **b** Effects of TP of different dosage on body weight in rats: Rats were orally treated with vehicle or 200, 400 or 600 μg/kg/day of TP for 28 days. Symbols and bars represent the mean ± SD, respectively; *P < 0.05, **P < 0.01 versus the control group. **c** Effects of TP of different dosage on liver index in rats showed no significant difference. **d-g** Pathological study of liver from the control group (**d**), 200 μg/kg/day (**e**), 400 μg/kg/day(**f**), or 600 μg/kg/day (**g**) for 28 days. **d** showed no remarkable changes; (**e-f**) showed apparent degeneration of liver cell, infiltration of inflammatory cells in hepatic lobules and portal area

After one-week administration of TP, the rats began to exhibit restlessness by jumping up and down. Some rats showed reduced spontaneous activity and were less responsive to outside stimulations. During the second week, the groups which were administered 400 and 600 μg/(kg.day) of TP began showing symptoms of mortality. In the third and fourth week, the rats of the TP groups showed several symptoms of severe poisoning such as arched vertical hair, squinting or closed eyes, reduced activity, diarrhea, weight loss, and death. Of the rats that lived through the 28-day treatment period, 11 were from the 200 μg/(kg.day) group, 8 were from the 400 μg/(kg.day) group, and 6 were from the 600 μg/(kg.day) group. The number of deaths observed during the 28 days is presented in (Fig. 2a).

#### Effects of TP on the body weight of rats

Before the treatment with TP, there was no significant difference in body weight among the four groups. After the 28-day treatment period, the body weight of TP-treated rats was markedly decreased (*P* < 0.05 or *P* < 0.01), especially during the latest week of measurement, compared with the steadily increasing trend observed in the control group. Among the TP treated groups, the group which received with 600 μg/(kg.day) of TP demonstrated a dramatic weight loss compared with that observed in other groups (*P* < 0.01). These experimental results are shown in (Fig. 2b).

#### Effects of TP on the liver index of rats

After the rats were sacrificed, the livers were placed on ice and weighed. The control group livers were dark red in color with bright red cut surfaces, soft and normal volumes, and smooth capsules. In contrast, the TP group livers were swollen with purple or dark cut surfaces. Based on the statistical analysis, there was no significant difference in liver index between the control and TP-treated groups (Fig. 2c).

#### Effects of TP on hepatic histopathology

Microscopically, the control group showed no major remarkable pathological changes. After the oral administration of TP for (200, 400, and 600 μg/(kg/.day)) for 28 days, the rats showed significant histopathological changes, mainly including apparent liver cell degeneration of liver cell and inflammatory cell infiltration of inflammatory cells in the hepatic lobules and portal area, as shown in (Fig. 2d-g).

#### Effects of TP on biochemical indicators

**Fig. 3:**
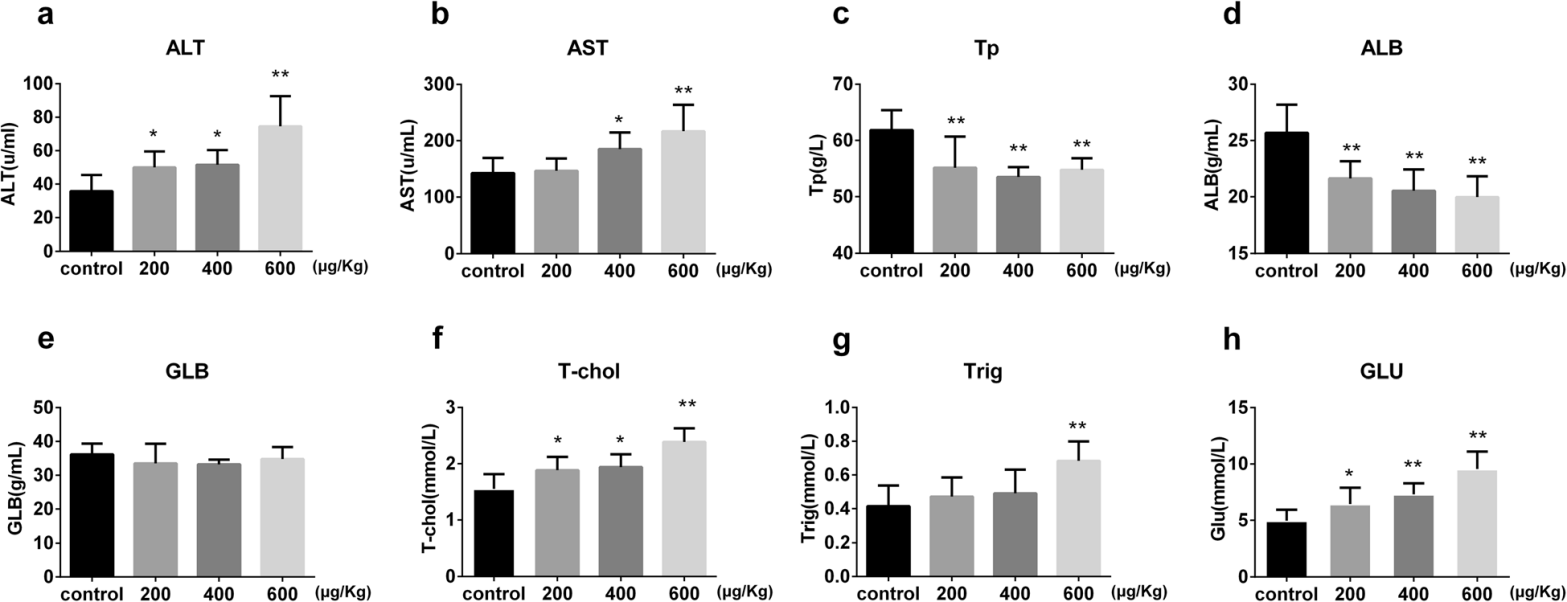
Effect of triptolide on biochemical indicators after orally treated with vehicle or 200, 400 or 600 μg/kg/day of triptolide for 28 days, the biochemical indicators exhibited significant increases of ALT (**a**), AST (**b**), T-chol (**f**), Trig (**g**). GLU (**l**), and significant decreases of Tp (**c**), ALB (**d**) were also observed. There shows no significant difference in GLB (**e**). Results are expressed as mean ± SD of each group, respectively;*P < 0.05, **P < 0.01 significantly different from the control group

As shown in Fig. 3, after the oral administration of either the vehicle or TP (200, 400, and 600 μg/(kg.day)) for 28 days, significant increase (*P* < 0.05 or *P* < 0.01) in the levels of ALT, AST, T-chol, Trig, and GLU and significant decrease (*P* < 0.05 or *P* < 0.01) in the levels of Tp and ALB were observed in the TP groups in comparison with that observed in the control group. Thus, most of the biochemical indicators changed in a TP dosage-dependent manner compared with the observed values in the control group. However, there was no significant difference in GLB levels (see Additional file 1).

### In vitro metabolic study

#### Effects of TP on rat liver microsomes

**Fig. 4:**
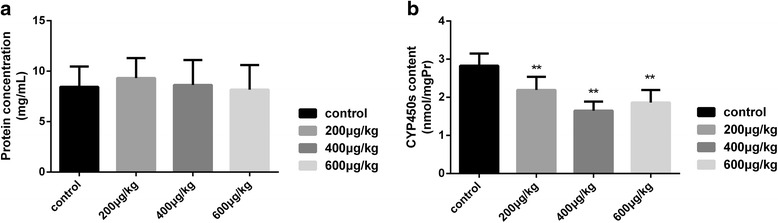
The effects of protein concentrations and CYP450s content of liver microsomes**. a** Effects of TP on the microsomal protein concentration showed no significant difference. **b** CYP450s content of liver microsomes showed a significant decrease (*P* < 0.01) after the TP administration of all the different dosages. Results are expressed as mean ± SD of each group, respectively;*P < 0.05, ** *P* < 0.01 significantly different from the control group

No significant difference in microsomal protein concentration was observed in TP group when compared to that in the control group (Fig. 4a). However, TP treatment caused a significant decrease (*P* < 0.01) in the microsomal CYP450 content (Fig. 4b), with the 400 μg/(kg.day) dose group exhibiting maximal suppression (58.3%) of CYP450 content.

#### Chromatography-mass behavior and specificity study

**Fig. 5:**
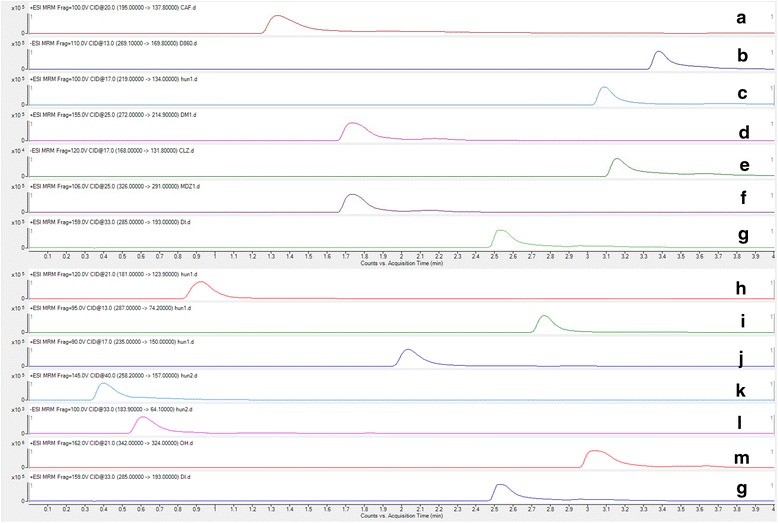
Chromatograms of substrates, metabolites and the internal standard. (*A*-*F*): Substrates[A: Caffeine(CAF);B: Tolbutamide(D860);C: (S)-Mephenytoin(MT);D: Dextromethorphan(DM);E:Chlorzoxazone(CLZ);F: Midazolam(MDZ)]. (*H*-*M*) metabolites [H:Paraxanthine;I:4-Hydroxy Tolbutamide;J:(+/−)-4-Hydroxy Mephenytoin;K:Dextrorphan;L:6-Hydroxy chlorzoxazone;M:1-Hydroxymidazolam]. (*G*) The internal standard: Diazepam(DZP)

Since our initial efforts were focused on monitoring the changes in CYP450 content upon oral administration of TP, we explored whether the six main CYP450 isoform-specific substrates and their metabolites were suitable for use in cocktail incubations. We thus established a cocktail assay for simultaneous determination of the activities of six main CYP450 isoforms in liver microsomes using UHPLC-MS/MS, with a cocktail probe consisting of the isoform-specific substrates, metabolites, and an internal standard. The multiple reaction monitoring (MRM) transitions, optimized fragmentor voltage, collision energy, and selected reaction monitoring (SRM) pairs of each substrate and its metabolite are reported in Table 3. The specificity of the tandem mass spectrometer allowed a fast liquid chromatography gradient to be employed. Representative chromatograms for substrates and metabolites in the microsomal incubation mixtures are presented in Fig. 5. There was no interference from other substrates or metabolites at any of the retention times of interest for any metabolite MRM channel.

**Table 3:** MRM chromatogram by UHPLC/MIS/MS method for each subject from a normal assay sample

component	Corresponding CYP450s	parent ion	daughter ion	Fragmentor /V	Collision energy/V	polarity
Caffeine(CAF)	CYP1A2	195.0	137.8	100	20	ESI+
Tolbutamide(D860)	CYP2C9	269.1	169.8	110	13	ESI-
(S)-Mephenytoin(MT)	CYP2C19	219.0	134.0	100	17	ESI+
Dextromethorphan(DM)	CYP2D6	272.0	214.9	155	25	ESI+
Chlorzoxazone(CLZ)	CYP2E1	168.0	131.8	120	17	ESI-
Midazolam(MDZ)	CYP3A	326.0	291.0	106	25	ESI+
Paraxanthine	CYP1A2	181.0	123.9	120	21	ESI+
4-Hydroxy Tolbutamide	CYP2C9	287.0	74.2	95	13	ESI+
(+/−)-4-Hydroxy Mephenytoin	CYP2C19	235.0	150.0	90	17	ESI+
Dextrorphan	CYP2D6	258.2	157.0	145.	40	ESI+
6-Hydroxy chlorzoxazone	CYP2E1	183.9	64.1	100	33	ESI-
1-Hydroxymidazolam	CYP3A	342.0	324	162	21	ESI+
Diazepam(DZP)	ISTD	285.0	193.0	159	33	ESI+

Stability tests showed that samples remained stable even after being placed at room temperature for 24 h and being subjected to three repeated freeze-thaw cycles. In the precision test, the average standard deviation of three substances corresponding to the peak area was < 5%. The recovery rate test showed that both the absolute and method recovery rates were > 90%.

#### Effects of TP on the substrates and metabolites of CYP450 enzymes

**Fig. 6:**
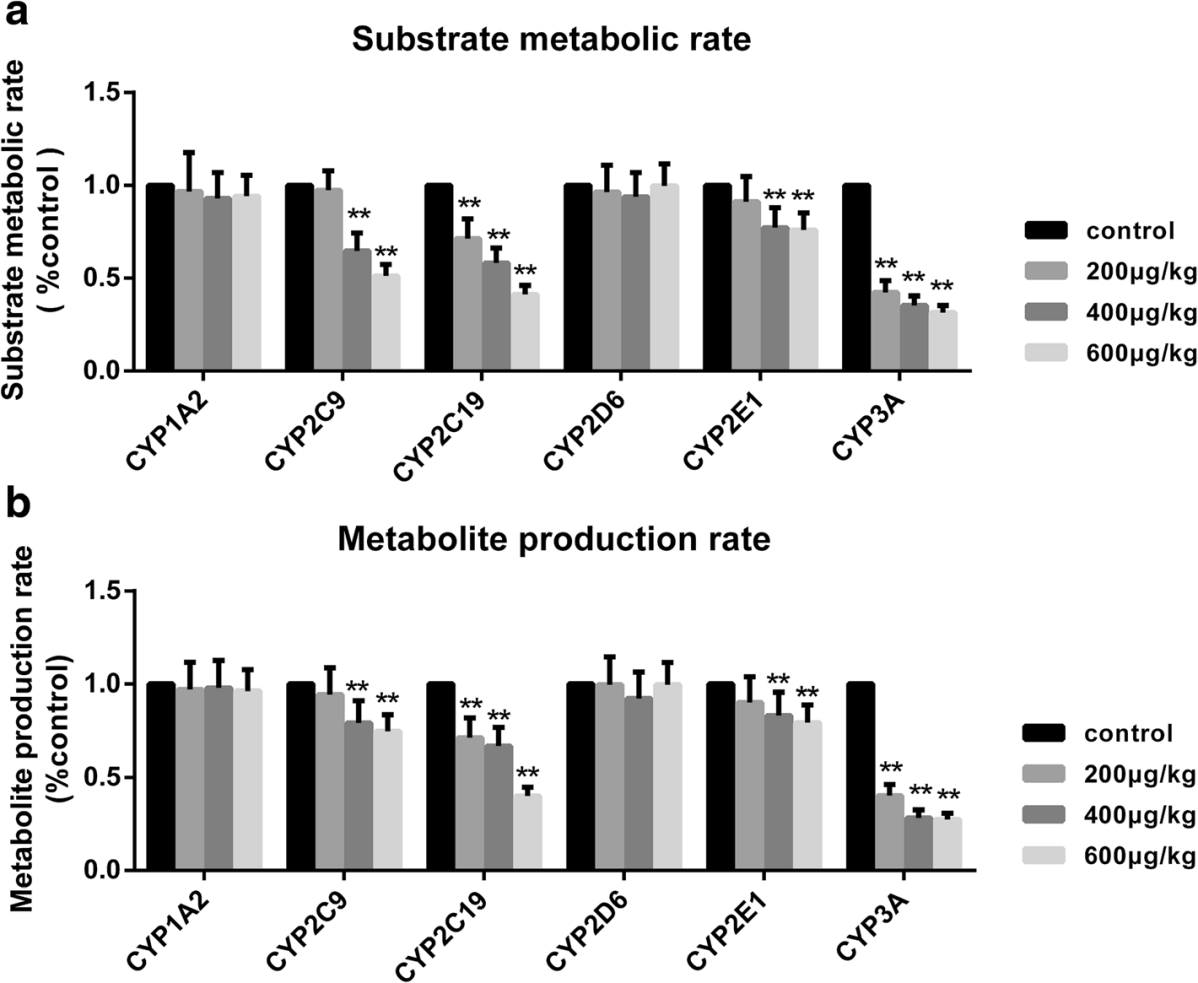
Effects of TP on the CYP450s enzyme activity. **a** The effect of TP on the substrate metabolic rate. After orally treated with vehicle or 200, 400 or 600 μg/kg/day of triptolide for 28 days, the liver microsomes were incubated with the cocktail probe (CAF/D860/MT/DM/CLZ/MDZ: 15/20/10/10/5/2.5 μM) for 30 mins. The final concentration of the substrates and metabolites were detected by UPLC-MS/MS. the substrate metabolic rate of CYP1A2, CYP2D6 after the 30-min-incubation in vitro remained the same but other CYP450s showed significant changes. The 200 μg/kg/day TP group exhibited decreases in CYP2C19 and CYP3A; then the 400 and 600 μg/kg/day TP group exhibited decreases not only in CYP2C19 and CYP3A, but also CYP2E1 and CYP2C9. **b** The effect of TP on the production of the metabolites. After the administration, the 200 μg/kg/day TP group showed decreases in CYP2C19 and CYP3A, then the 400 and 600 μg/kg/day TP group also exhibited decreases in CYP2E1 and CYP2C9. Results are expressed as mean ± SD of each group, respectively;*P < 0.05, ** *P* < 0.01 significantly different from the control group

In this study we determined the enzyme activity index in terms of the substrate metabolic rate and the metabolite production rate [nmol/(protein g × min)]. After oral administration of either the vehicle or TP (200, 400 or 600 μg/(kg.day)) for 28 days, the liver microsomes were incubated with the cocktail probe (CAF/D860/MT/DM/CLZ/MDZ: 15/20/10/10/5/2.5 μM) for 30 min. The final concentrations of the substrates and metabolites were detected by UHPLC-MS/MS. Although the metabolic rates of CYP1A2 and CYP2D6 remained the same, the rates of other CYP450s showed significant changes (*P* < 0.05 or *P* < 0.01) in TP-treated groups than in the control group (Fig. 6a). While the metabolic rates of CYP2C19 (71.3%) and CYP3A (40.2%) decreased in the 200 μg/(kg.day) TP group, the rates of CYP2C19 (58.1%), CYP3A (35.4%), CYP2E1 (77.2%), and CYP2C9 (64.6%) were decreased in the 400 μg/(kg.day) TP group. Similarly, the 600 μg/(kg.day) TP group showed a more dramatic decrease in the rates of CYP2C19 (41.1%), CYP3A (31.6%), CYP2E1 (75.9%), and CYP2C9 (51.1%). Similar decreases (*P* < 0.05 or *P* < 0.01) were also seen in the metabolite production rates in TP-treated groups in comparison with that observed in the control group (Fig. 6b). While the metabolite production rates of CYP2C19 (70.1%) and CYP3A (40.2%) decreased in the 200 μg/(kg.day) TP group, the rates of CYP2C19 (58.1%), CYP3A (35.4%), CYP2E1 (66.7%), and CYP2C9 (28.1%) decreased in the 400 μg/(kg.day) TP group. The 600 μg/(kg.day) TP group showed a more dramatic decrease in the metabolite production rates of CYP2C19 (41.1%), CYP3A (31.6%), CYP2E1 (39.8%), and CYP2C9 (27.4%) (See Additional file 2).

Thus, when compared with the control group, 28-day TP administration inhibited either the substrate reduction or metabolite production. Further, decrease in the activities of CYP450s became more pronounced with the increase in TP dosage.

#### Effects of TP on the kinetic parameters of CYP450 family

**Fig. 7:**
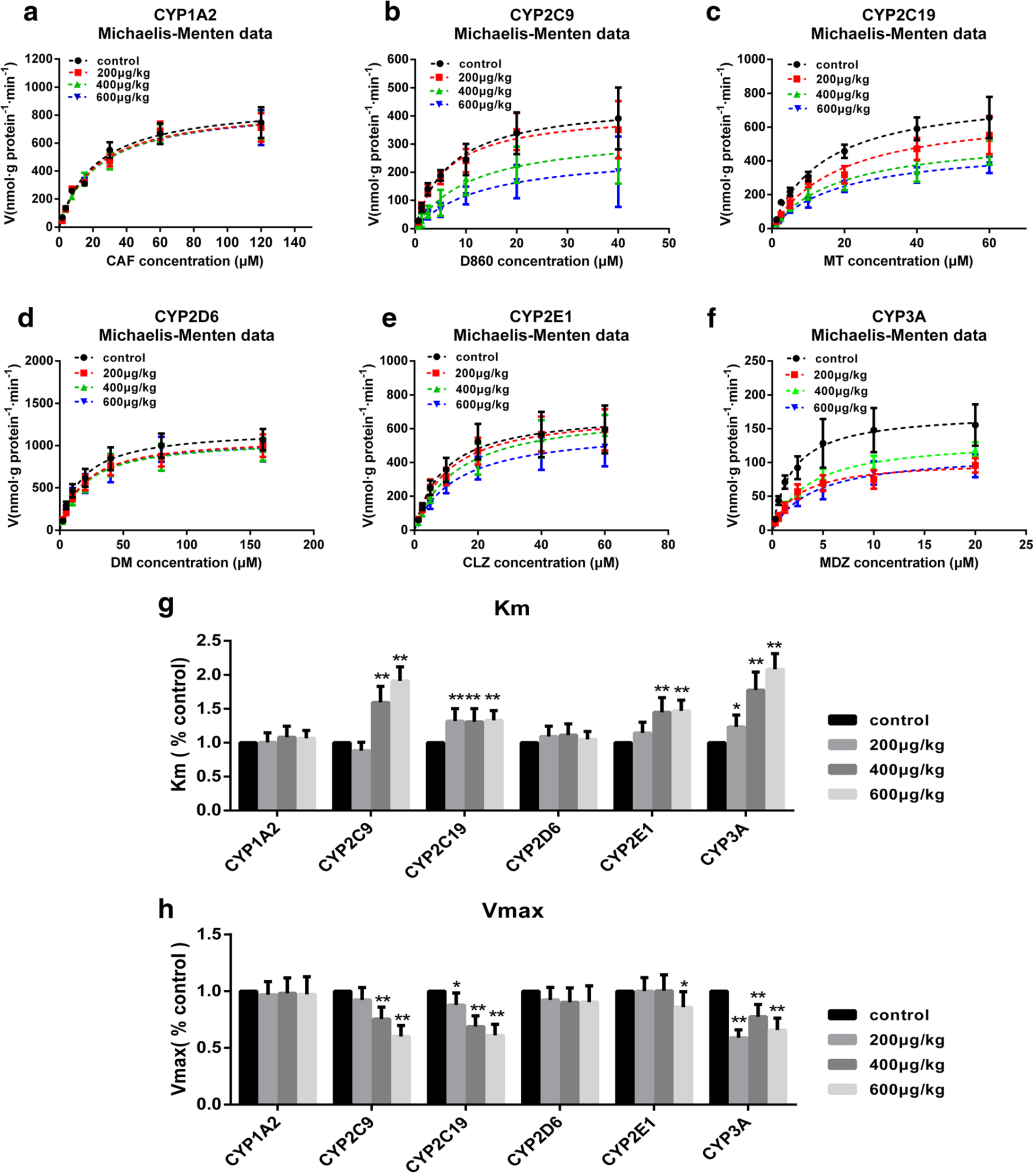
Effects of TP on the CYP450s kinetic parameters. **a**-**f** The Michaelis–Menten data of each CYP450s. After orally treated with vehicle or 200, 400 or 600 μg/kg/day of triptolide for 28 days, the liver microsomes incubated with the cocktail probe (CAF/D860/MT/DM/CLZ/MDZ) in different concentration (diluted with half dilution method as listed in Table 1) and a NADPH-generating system at 37 °C. The concentration of the each metabolites were detected by UPLC-MS/MS at the incubation time of 1, 2, 4, 6, 10 min, the rate of metabolism were calculated from linear regression analysis. The rate of metabolism [nmol/(protein g × min)] versus the concentration of each substrates was fit to The Michaelis–Menten equation. Data represents the mean of three independent experiments in duplicate determinations. **g-h** The Km (g) and Vmax values (h) were calculated from non-linear regression analysis of experimental data according to the Michaelis–Menten models for the kinetics of each CYP450s with Prism 5.0 program. Results are expressed as mean ± SD of each group, respectively;*P < 0.05, ** *P* < 0.01 significantly different from the control group

In this study, we observed the production of the metabolites [nmol/(protein g × min)] by incubating the microsomes with different concentrations of the substrates. The Km and Vmax values of each CYP450 isoform were calculated by non-linear regression analysis of experimental data according to the Michaelis–Menten model of enzyme kinetics, using the Prism 5.0 program. As shown in Fig. 7, after oral administration of either the vehicle or TP (200, 400 or 600 μg/(kg.day)) for 28 days, the liver microsomes were incubated with different concentrations of the cocktail probe (CAF/D860/MT/DM/CLZ/MDZ) for 1, 2, 4, 6, and 10 min. In the TP-treated groups, the Km values of CYP1A2 and CYP2D6 remained the same while the corresponding values of other CYP450s showed significant changes (*P* < 0.05 or *P* < 0.01), in comparison with the control group values. The 200 μg/(kg.day) TP group exhibited an increase in the Km values of CYP2C19 (1.31 fold) and CYP3A (1.23 fold). Similarly, the 400 μg/(kg.day) TP group exhibited an increase in the Km values of CYP2C19 (1.30 fold), CYP3A (1.78 fold), CYP2E1 (1.45 fold), and CYP2C9 (1.59 fold). The 600 μg/(kg.day) TP group showed a more dramatic increase in the Km values of CYP2C9 (1.91 fold), CYP3A (2.08 fold), CYP2E1 (1.47 fold), and CYP2C19 (1.33 fold). The metabolite production rates followed a decreasing trend (*P* < 0.05 or *P* < 0.01) in the TP-treated groups in comparison to that observed in the control groups. While the metabolite production rates of CYP2C19 (87.8%) and CYP3A (58.8%) were decreased in the 200 μg/(kg.day) TP group, the rates of CYP2C19 (68.7%), CYP3A (77.5%), and CYP2C9 (75.4%) were decreased in the 400 μg/(kg.day) TP group. The 600 μg/(kg.day) TP group showed a more dramatic decrease in the metabolite production rates of CYP2C19 (60.9%), CYP3A (65.7%), CYP2C9 (60.0%), and CYP2E1 (85.9%) (see Additional file 3).

The Km value represents the affinity of a CYP450 enzyme for its specific substrate while the Vmax value represents the theoretical maximum metabolic rate of the substrate in the incubation system. These two parameters are very important indicators in the study of the kinetics of enzyme-catalyzed reactions. In comparison with the Km and Vmax value trends of CYP450s in the control groups, the trends observed in the TP-treated groups after the 28-day treatment period reflected the inhibitory effects of TP on CYP450 activity. Further, this TP-induced inhibition of CYP450 activity became more pronounced with the increase in TP dosage.

### Verification in mRNA and protein level

#### Effect on mRNA expression of CYP3A and CYP2C9

**Fig. 8:**
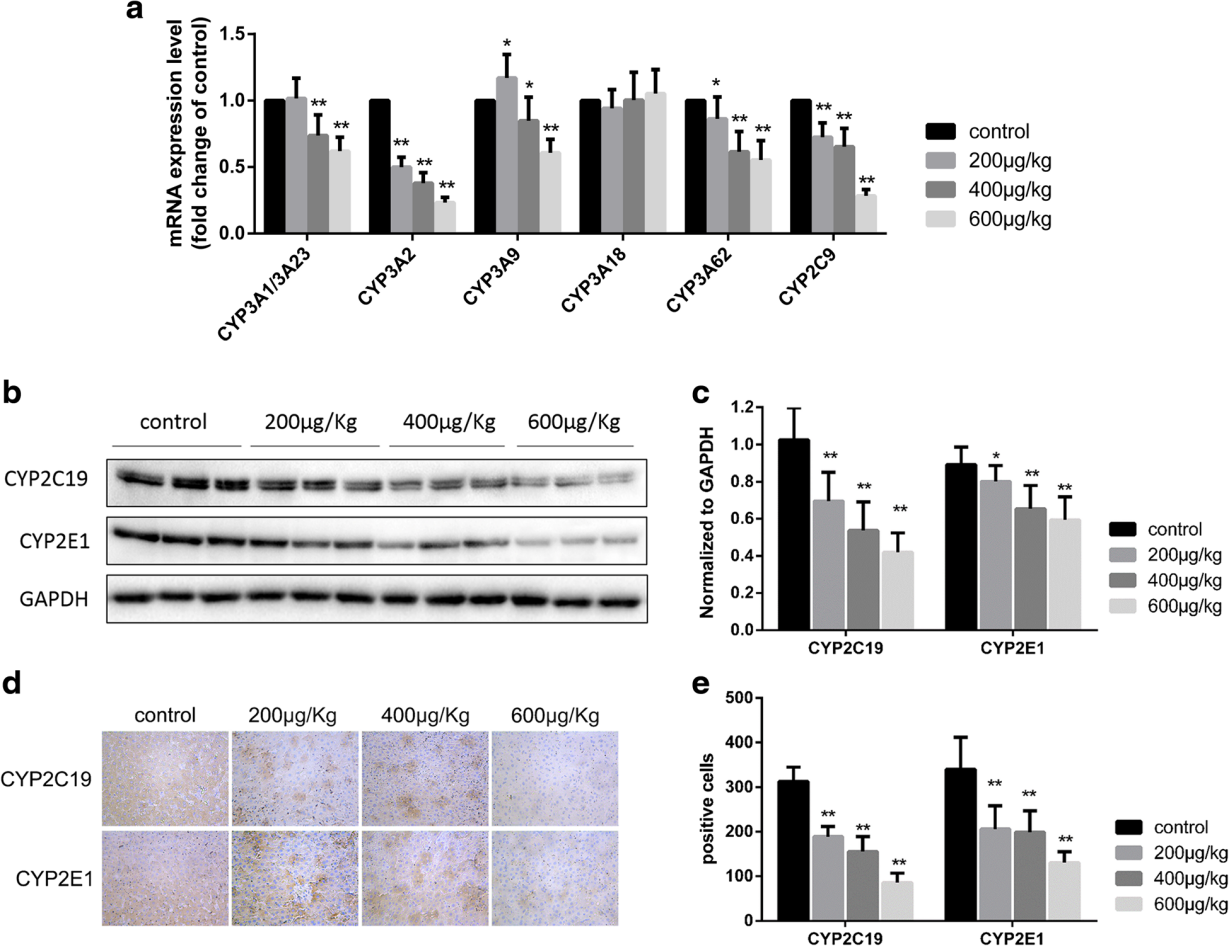
Verification in mRNA and protein level. **a** Effect of triptolide on mRNA levels of CYP3A family and CYP2C9 in livers of rats. Liver lysates were made from livers of rats. **b** Protein lysates were made from microsomes of rats’ liver and subjected to Western blot analysis using the indicated antibodies. The GAPDH was used as an internal control. A representative blot from three rats in each group was shown. The density of the immunoreactive bands was analyzed as(**c**). **d** Immunohistochemical detection of CYP2C19 and CYP2E1 in livers section of rats was shown. The Immunoreactive cells were stereo logically quantified (**e**). Symbols and bars represent the mean ± SD, respectively; *p < 0.05, **p < 0.01 versus the control group

After oral administration of either the vehicle or TP (200, 400 or 600 μg/(kg.day)) for 28 days, the mRNA levels of many CYP3A subfamily isoforms and CYP2C9 in TP-treated groups showed a significant decrease (*P* < 0.05 or *P* < 0.01) in comparison with that observed in the control group (Fig. 8a). The 200 μg/(kg.day) TP group exhibited decreased CYP3A2 (49.8%) and CYP3A62 (86.2%), and increased CYP3A9 (1.17 fold) mRNA expression. The 400 μg/(kg.day) TP group also exhibited decreased CYP3A2 (37.8%), CYP3A62 (61.5%), CYP3A1/3A23 (73.7%), and CYP3A9 (84.8%) mRNA expression. The 600 μg/(kg.day) TP group exhibited a stronger decrease in CYP3A1/3A23 (61.8%), CYP3A2 (23.2%), CYP3A9 (60.6%), and CYP3A62 (55.2%) mRNA expression. Expression of CYP2C9 mRNA in the 200, 400, and 600 μg/(kg.day) TP groups decreased in a dose-dependent manner to 72.3, 66.3, and 28.2%, respectively, in comparison with that observed in the control.

#### Effect on protein expression of CYP2C19 and CYP2E1

When the effects of TP on CYP450 protein expression in rat liver microsomes were studied (Fig. 8b-c), TP was observed to decrease the protein expression of CYP2C19 and CYP2E1 in a dose-dependent manner (*P* < 0.05 or *P* < 0.01), in comparison to that observed in the control. The 600 μg/(kg.day) TP group exhibited a particularly strong decrease in CYP2C19 (41.9%) and CYP2E1 (51.4%) protein expression.

Results of the immunohistochemical detection of CYP2C19 and CYP2E1 in rat liver sections were as shown in (Fig. 8d-e). TP was observed to decrease CYP2C19 and CYP2E1 expression in immunoreactive cells in a dose-dependent manner (*P* < 0.05 or *P* < 0.01), in comparison to that observed in the control. The immunoreactive cells of the 600 μg/(kg.day) TP group exhibited a particularly strong decrease in CYP2C19 (27.5%) and CYP2E1 (38.2%) expression.

## Discussion

Based on pre-experimental observations, we found that TP-induced liver injury was more severe and stable in female rats than in male rats. Hence, female SD rats were specifically selected for this study. We found a certain correlation between the dosages of TP and hepatotoxicity in rats, which was consistent with previously published results [7, 21]. The TP-treated groups exhibited histopathological changes. Additionally, changes in the biochemical indicators suggested that the mechanisms of liver injury differed with different TP dosages and would be progressed by higher concentration.

An intensive understanding of the CYP450 dysregulation phenomenon in TP-induced liver injury is necessary because of its significance in guiding the drug dosage in patients who require the co-administration of other drugs with TP. As shown in Fig. 4, TP decreased the activities of CYP2C19, CYP2C9, CYP2E1 and the CYP3A subfamily without attenuating the liver microsomal protein concentration. CYP3A, which metabolizes 60% of all clinical drugs, is the most important enzyme for drug metabolism. In our study, the result about CYP3A confirmed its major role in the metabolism of TP. It should be noted that drugs which are metabolized by CYP3A should be avoided or reasonably adjusted when co-administered with TP or its related preparations, orally. The metabolic activation of carcinogens and toxins is mainly mediated by CYP2E1 [29, 30], and drugs of the nervous system are mainly metabolized by the CYP2C subfamily [31, 32]. Clinicians should therefore closely monitor the patient’s liver-related indices and adjust the medication when co-administering drugs which are mainly metabolized by these CYP450s along with TP to avoid toxic side effects.

In our study, we observed a TP dose-dependent decrease in the mRNA expression of most CYP3A subfamily isoforms and CYP2C9, which was consistent with the results of our metabolic study. Among the five main CYP3A isoforms in rats, the expression of CYP3A2 and CYP3A62 mRNAs was most sensitive to TP-induced toxicity. The expression of CYP3A1/3A23 and CYP3A9 mRNAs was also significantly decreased at higher TP dosages, while there was no such inhibitory effect on CYP3A18. This phenomenon may have been due to the use of adult rats in this experiment, as CYP3A18 expression is strongly correlated with growth hormone production [33]. CYP3A4 was reported to be primarily responsible for the hydroxylation of TP in the human liver [16]. In vitro studies of TP with CYP isoforms and P-glycoprotein (P-gp) indicate that TP has the potential to cause pharmacokinetic drug interactions when co-administered with other drugs metabolized by CYP1A2 and CYP3A4 [34]. As the major member of CYP3A subfamily in rats, CYP3A1/3A23 and CYP3A2 had 89% in their amino acid sequences [35], and CYP3A2 was reported to be specifically expressed in male rats [36]. In this study, we found that CYP3A2 played a very important role in the dysregulation of CYP450 activity due to TP-induced liver injury, in female rats as well. In spite of the sequence similarity, many functional differences exist between CYP3A1/3A23 and CYP3A2. [28]. In our study, while the mRNA expression pattern of CYP3A62 and CYP3A9 showed similar trends in the 400 and 600 μg/(kg.day) TP groups, the trends were completely different in the 200 μg/(kg.day) TP group. This phenomenon may have been due to the metabolic difference exhibited by different concentrations of TP and needs to be investigated further.

CYP2C9, an important component of the liver enzyme system, comprises about 10% of the total liver CYP450 enzyme content and is involved in the metabolism of 10% of the drugs in clinical trials [37]. In this study, we observed that CYP2C9 activity in the 200 μg/(kg.day) TP group was not inhibited although its mRNA expression was significantly decreased, after the administration of TP for 28 days. But in the 400 and 600 μg/(kg.day) TP groups, the enzyme affinity, activity, and mRNA expression of CYP2C9 were significantly decreased, thus confirming the dose-dependent inhibitory effect of TP on CYP2C9.

According to our results, the protein expression of CYP2C19 and CYP2E1 decreased in a TP dose-dependent manner, which was consistent with the results of our metabolic study. Besides the CYP3A subfamily, CYP2C19 is also reported to be involved in TP metabolism and toxicity [16, 34], and is therefore being actively researched. High doses of TP have been previously reported to significantly upregulate the levels of CYP2E1 mRNA in comparison with that observed in the control, after intragastric administration of TP (0.05, 0.3 and 0.6 mg/(kg.day)) for 7 consecutive days [38]. However, in our study, we observed a significant decrease in CYP2E1 enzyme affinity and activity in TP-treated groups, and the protein expression study further confirmed this inhibitory effect of TP. We speculate that the difference between our findings and those of the previous report [38] may be due to a difference in the time of intragastric administration of TP.

This experimental research demonstrated the abnormal metabolism of CYP450 enzyme system after TP administration. This results will hopefully serve as useful information for further understanding of the underlying mechanisms of TP-induced subacute toxicity. This results also suggests that when the patient needs to take TP for a certain time, doctors should pay attention to avoid the co-administration with drugs which metabolized by CYP3A, CYP2C9, CYP2C19, CYP2E1. If the co-administration was inevitable, doctors should closely observe the liver function.

There are several limitations in our work. First, the relationship between CYP450 dysregulation and TP-induced hepatotoxicity remains undefined because we did not determine the exact mechanism through which TP affects CYP450 activity. Second, although we observed the effect of different concentrations of TP on CYP450 activity, the effect of different times of drug administration still needs to be investigated. Finally, all our experiments were conducted in rat liver microsomes or tissues; therefore, the effects of TP on real patients need to be investigated in the future.

## Conclusions

In summary, intragastric administration of TP (200, 400, and 600 μg/(kg.day)) for 28 consecutive days can cause hepatotoxicity by reducing the substrate affinity, activity, and expression at the transcriptional and protein level of CYP3A, CYP2C9, CYP2C19, and CYP2E1. TP also has the potential to cause pharmacokinetic drug interactions when co-administered with drugs metabolized by these four CYP450 isoforms. However, further clinical studies are needed to evaluate the significance of this interaction.

## Additional files

Additional file 1: Table S1. Effect of TP on Biochemical Indicators. (DOCX 14 kb)
Additional file 2: Table S2. Effects of TP on the CYP450s enzyme substrates and metabolites. (DOCX 16 kb)
Additional file 3: Table S3. Effects of TP on the CYP450s kinetic parameters. (DOCX 15 kb)

## Abbreviations

ALB: Albumin
ALT: Alanine aminotransferase
AST: Aspartate aminotransferas
CAF: Caffeine
CLZ: Chlorzoxazone
CYP450s: Cytochrome P450 enzymes
D860: Tolbutamide
DDI: Drug–drug interaction
DM: Dextromethorphan
DTT: Dithiothreitol
DZP: Diazepam
EDTA: Ethylene Diamine Tetraacetic Acid
GLB: Globulin
Glu: Glucose
ISTD: Internal standard
MDZ: Midazolam
mRNA: Messenger Ribonucleic Acid
MT: (S)-Mephenytoin
PAGE: Polyacrylamidegel electrophoresis
PMSF: Phenylmethanesulfonyl fluoride
PVDF: Poly-vinylidene difluoride
RIPA: Radio immuno precipitation
RT-qPCR: Quantitative real-time polymerase chain reaction
SDS: Sodium dodecylsulphate
T-chol: Total cholesterol
TG: Triglycerides
TPT: riptolide
Tp: Total protein
TWHF: Tripterygium wilfordii Hook F
UPLC-MS/MS: Ultra performance liquid chromatography-tandem mass spectrometry.
