# Supplementary material for: Triptolide Induces hepatotoxicity via inhibition of CYP450s in Rat liver microsomes
Source: BMC Complement Altern Med. 2017 Jan 5;17:15. doi: 10.1186/s12906-016-1504-3 (PMC5217299; doi:10.1186/s12906-016-1504-3)
Supplement: Additional file 1: Table S1. — Effect of TP on Biochemical Indicators. (DOCX 14 kb) [file 12906_2016_1504_MOESM1_ESM.docx]

Table S1 Effect of TP on Biochemical Indicators

| Parameter | Dose of TP (μg/kg/day) | | | |
| --- | --- | --- | --- | --- |
|  | 0 | 200 | 400 | 600 |
| N | 12 | 11 | 8 | 6 |
| ALT（u/mL） | 35.75±9.54 | 50.00±9.53* | 51.63±8.73* | 74.67±17.99** |
| AST（u/mL） | 142.3±27.0 | 146.5±21.7 | 185.0±29.6* | 216.8±46.7** |
| Tp（g/L） | 61.82±3.38 | 55.16±5.54** | 53.51±1.76** | 54.78±2.04** |
| ALB（g/L） | 25.68±2.57 | 21.65±1.52** | 20.55±1.88** | 19.98±1.85** |
| G（g/L） | 36.14±2.58 | 33.51±5.81 | 32.96±0.87 | 34.80±3.56 |
| T-chol（mmol/L） | 1.550±0.226 | 1.881±0.235* | 1.943±0.226* | 2.388±0.243** |
| TG（mmol/L） | 0.415±0.123 | 0.485±0.112 | 0.491±0.140 | 0.683±0.116** |
| GLU（mmol/L） | 4.952±0.922 | 6.438±1.449* | 6.310±1.054** | 9.551±1.557** |

Biochemical Indicators after orally treated with vehicle or 200, 400 or 600 μg/kg/day of TP for 28 days (Mean±SD).

* *P* < 0.05 significantly different from the control.

** *P* < 0.01 significantly different from the control.
