# Supplementary material for: Triptolide Induces hepatotoxicity via inhibition of CYP450s in Rat liver microsomes
Source: BMC Complement Altern Med. 2017 Jan 5;17:15. doi: 10.1186/s12906-016-1504-3 (PMC5217299; doi:10.1186/s12906-016-1504-3)
Supplement: Additional file 2: Table S2. — Effects of TP on the CYP450s enzyme substrates and metabolites. (DOCX 16 kb) [file 12906_2016_1504_MOESM2_ESM.docx]

Table S2 Effects of TP on the CYP450s enzyme substrates and metabolites

|  |  | N | Final concentration of substrate(μg /l) | Final concentration of metabolites(μg /l) | Substrate metabolic rate (%) | Metabolite production rate  [nmol/ (protein g × min)] |
| --- | --- | --- | --- | --- | --- | --- |
| 1A2 | Control | 12 | 1.354±0.341 | 6.983±1.758 | 53.44±8.01 | 534.4±101.7 |
|  | 200μg/kg | 11 | 1.351±0.281 | 6.982±1.448 | 51.69±8.34 | 518.6±97.5 |
|  | 400μg/kg | 8 | 1.462±0.324 | 7.552±1.655 | 49.65±7.44 | 523.0±148.9 |
|  | 600μg/kg | 6 | 1.444±0.322 | 7.448±1.655 | 50.34±7.55 | 514.0±123.4 |
| 2C9 | control | 12 | 0.368±0.084 | 1.326±0.296 | 73.48±8.82 | 314.7±29.5 |
|  | 200μg/kg | 11 | 0.411±0.092 | 1.456±0.341 | 71.46±8.16 | 297.1±31.4 |
|  | 400μg/kg | 8 | 0.709±0.365** | 2.626±1.111** | 47.48±5.70** | 249.4±44.4** |
|  | 600μg/kg | 6 | 0.842±0.145** | 3.120±0.519** | 37.59±4.51** | 235.3±77.3** |
| 2C19 | control | 12 | 0.491±0.122 | 2.247±0.550 | 77.52±9.30 | 765.3±97.1 |
|  | 200ug/kg | 11 | 0.851±0.242** | 3.899±1.100** | 61.00±7.32** | 545.4±76.3** |
|  | 400ug/kg | 8 | 0.986±0.387** | 4.495±1.743** | 45.05±6.60** | 511.2±71.5** |
|  | 600uo/kg | 6 | 1.055±0.324** | 4.816±1.467** | 31.83±6.22** | 305.3±88.6** |
| 2D6 | control | 12 | 4.320±1.333 | 11.67±3.59 | 41.62±9.30 | 1294±215 |
|  | 200μg/kg | 11 | 4.437±1.555 | 11.97±4.18 | 40.14±8.26 | 1415±211. |
|  | 400μg/kg | 8 | 4.661±1.042 | 12.59±2.81 | 39.03±7.32 | 1196±218 |
|  | 600μg/kg | 6 | 4.332±1.551 | 11.70±4.18 | 41.49±7.32 | 1288±207 |
| 2E1 | control | 12 | 0.917±0.125 | 5.352±0.705 | 46.47±5.11 | 134.2±25.6 |
|  | 200μg/kg | 11 | 0.981±0.214 | 5.764±1.235 | 42.35±4.65 | 121.1±16.5 |
|  | 400μg/kg | 8 | 1.094±0.247** | 6.411±1.411** | 35.88±3.94** | 111.6±27.8** |
|  | 600μg/kg | 6 | 1.154±0.252** | 6.470±1.470** | 35.29±3.88** | 106.5±34.7** |
| 3A | control | 12 | 4.324±1.335 | 0.879±0.219 | 64.83±12.96 | 165.4±39.6 |
|  | 200μg/kg | 11 | 4.434±1.557** | 1.565±0.247** | 27.36±7.47** | 66.47±41.44** |
|  | 400μg/kg | 8 | 4.664±1.041** | 1.675±0.879** | 22.96±6.59** | 46.67±15.64** |
|  | 600μg/kg | 6 | 4.334±1.557** | 1.538±0.467** | 20.46±7.69** | 45.34±14.54** |

After orally treated with vehicle or 200, 400 or 600 μg/kg/day of TP for 28 days, the liver microsomes were incubated with the cocktail probe (CAF/D860/MT/DM/CLZ/MDZ: 15/20/10/10/5/2.5 μM) for 30 mins. The final concentration of the substrates and metabolites were detected by UHPLC-MS/MS. Results are expressed as mean ± SD

* *P* < 0.05 significantly different from the control.

** *P* < 0.01 significantly different from the control.
