# Supplementary material for: Triptolide Induces hepatotoxicity via inhibition of CYP450s in Rat liver microsomes
Source: BMC Complement Altern Med. 2017 Jan 5;17:15. doi: 10.1186/s12906-016-1504-3 (PMC5217299; doi:10.1186/s12906-016-1504-3)
Supplement: Additional file 3: Table S3. — Effects of TP on the CYP450s kinetic parameters. (DOCX 15 kb) [file 12906_2016_1504_MOESM3_ESM.docx]

Table S3 Effects of TP on the CYP450s kinetic parameters

|  |  | Dose of TP (μg/kg/day) | | | |
| --- | --- | --- | --- | --- | --- |
|  |  | 0 | 200 | 400 | 600 |
|  | N | 12 | 11 | 8 | 6 |
| 1A2 | Vmax[nmol/ (protein g × min)] | 895.2±25.78 | 866.8±26.59 | 878.4±30.21 | 870.2±38.87 |
|  | Km(μM) | 21.63±1.769 | 21.71±1.89 | 23.37±2.233 | 22.99±2.867 |
| 2C9 | Vmax[nmol/ (protein g × min)] | 452.8±23.14 | 417.5±20.51 | 341.4±37.37** | 271.7±48.98** |
|  | Km(μM) | 6.98±1.769 | 6.152±0.8977 | 11.12±3.042** | 13.33±5.677** |
| 2C19 | Vmax[nmol/ (protein g × min)] | 807.3±28.25 | 709.1±37.55* | 554.7±34.13** | 492.2±31.68** |
|  | Km(μM) | 14.66±1.4 | 19.33±2.551** | 19.15±2.945** | 19.46±3.113** |
| 2D6 | Vmax[nmol/ (protein g × min)] | 1196±32.75 | 1104±31.45 | 1079±41.94 | 1080±49.07 |
|  | Km(μM) | 17.08±1.531 | 18.62±1.698 | 18.99±2.349 | 17.91±2.63 |
| 2E1 | Vmax[nmol/ (protein g × min)] | 706.3±31.99 | 706.2±29.34 | 709.1±39.27 | 606.5±40.29* |
|  | Km(μM) | 9.276±1.317 | 10.59±1.327 | 13.43±2.087** | 13.61±2.527** |
| 3A | Vmax[nmol/ (protein g × min)] | 174.5±7.005 | 102.6±3.59** | 135.2±5.122** | 114.6±6.875** |
|  | Km(μM) | 1.994±0.2666 | 2.46±0.2713* | 3.543±0.3833** | 4.153±0.6795** |

After orally treated with vehicle or 200, 400 or 600 μg/kg/day of TP for 28 days, the liver microsomes incubated with the cocktail probe (CAF/D860/MT/DM/CLZ/MDZ) in different concentration (diluted with half dilution method as listed in Table 1) and a NADPH-generating system at 37°C. The concentration of the each metabolites were detected by UHPLC-MS/MS at the incubation time of 1, 2, 4, 6, 10 min, the rate of metabolism were calculated from linear regression analysis. The rate of metabolism [nmol/ (protein g × min)] versus the concentration of each substrates was fit to the Michaelis–Menten equation. Results are expressed as mean ± SD.

* *P* < 0.05 significantly different from the control.

** *P* < 0.01 significantly different from the control.
